# P-645. The Disease Burden of Respiratory Syncytial Virus-Related Hospitalization in Taiwanese Adults: A National Health Insurance Database Study

**DOI:** 10.1093/ofid/ofaf695.858

**Published:** 2026-01-11

**Authors:** Chee-Jen Chang, Hsiao-Ting Juang, Wei-Ting Chen, Kai-Ting Chen, Yu-Chen Kuo, Hazel Huang, Yhu-Chering Huang

**Affiliations:** Chang Gung University, Taoyuan, Taoyuan, Taiwan; Chang Gung University, Taoyuan, Taoyuan, Taiwan; MSD Taiwan, Taipei, Taipei, Taiwan; MSD Taiwan, Taipei, Taipei, Taiwan; Oracle Life Sciences, Taipei, Taipei, Taiwan; Oracle Life Sciences, Taipei, Taipei, Taiwan; Chang Gung Memorial Hospital, Chang Gung University, College of Medicine, Taoyuang, Taoyuan, Taiwan

## Abstract

**Background:**

Respiratory Syncytial Virus (RSV) remains a major cause of respiratory tract infections among older adults, especially among the elderly and those with comorbidities. This study investigated the incidence and disease burden of RSV-related hospitalization in Taiwanese adults.

**Methods:**

Our retrospective study was conducted among adults aged ≥ 18 years utilizing the National Health Insurance Research Database between July 2017-December 2021. Cases were defined as having ≥ 1 RSV-related hospitalization via the corresponding ICD-10 disease codes. The incidence, severity, risk factors, and medical costs related to RSV hospitalizations were examined.

**Results:**

Among 226 RSV-related hospitalizations, 66.8% occurred in older adults aged ≥ 60 years. Of these, 39.4% were aged ≥ 75 years. There was a 4-fold increase in the incidence of RSV-related hospitalizations in Q4 2020 (0.20 per 100,000 population) compared to 2017-2019 (0.05-0.07). Mean of hospitalization was 32 days, with 63.3% of cases requiring oxygen supplementation, 26.5% with intensive care unit (ICU) admission, and 23.0% with ventilator use. Among comorbid patients, congestive heart failure (CHF)/coronary artery disease (CAD)/stroke and chronic kidney disease were 2 times more reported to be admitted into the ICU (odds ratio, OR: 2.50; p=0.01 and 2.28; p=0.04 respectively). Risk factors for infection severity included being adults aged ≥ 50 years (ref: age 18-49 years; OR: 3.14-8.90, p< 0.05), having asthma/chronic obstructive pulmonary disease (COPD) (3.69; p< 0.05), and CHF/CAD/stroke (3.20; p< 0.05). Patients aged ≥ 75 years were the highest risk group for CHF/CAD/stroke (55.7%) and asthma/COPD (45.5%) for RSV-related hospitalizations. Mean medical costs were 9,209 USD for hospitalization, 4,786 USD for ICU admission, 2,720 USD for outpatient visit, and 259 USD for emergency room visits.

**Conclusion:**

Our study reveals the underestimated burden of RSV-related hospitalizations among Taiwanese adults, particularly those aged ≥ 50 and with comorbidities. The findings underscore the importance of RSV-vaccination and early intervention, especially among high-risk groups. Efforts should prioritize reducing the clinical and financial burden of RSV.

**Disclosures:**

Wei-Ting Chen, MD, MSD Taiwan: Employment Kai-Ting Chen, PhD, MSD Taiwan: Employment

